# Supplementary material for: Perceived Social Support Mediates the Relationship between Use of Greenspace and Geriatric Depression: A Cross-Sectional Study in a Sample of South-Italian Older Adults
Source: Int J Environ Res Public Health. 2023 Apr 17;20(8):5540. doi: 10.3390/ijerph20085540 (PMC10138493; doi:10.3390/ijerph20085540)
Supplement: Supplementary file 1 [file ijerph-20-05540-s001.zip › ijerph-2327641-supplementary.pdf]

Table S1. Scale Reliability Statistics of the Duke-UNC Functional Support Scale Questionnaire

	Mean	Sd	Cronbach's α	McDonald's ω	Skewness	Kurtosis
**scale**	3.84	0.869	0.842	0.846	-0.619	-0.400

Table S2. Item Reliability Statistics of the Duke-UNC Functional Support Scale Questionnaire

	Mean	Sd	Item-rest correlation	Cronbach's α	McDonald's ω	Skewness	Kurtosis
**Item 1**	4.04	1.15	0.553	0.826	0.830	-1.09	0.380
**Item 2**	4.19	1.05	0.564	0.826	0.830	-1.35	1.27
**Item 3**	3.78	1.28	0.655	0.813	0.819	-0.727	-0.673
**Item 4**	3.82	1.29	0.680	0.810	0.815	-0.843	-0.433
**Item 5**	3.60	1.45	0.657	0.813	0.819	-0.646	-0.994
**Item 6**	3.37	1.40	0.445	0.842	0.844	-0.354	-1.17
**Item 7**	3.65	1.29	0.594	0.821	0.828	-0.608	-0.760
**Item 8**	4.30	1.12	0.475	0.835	0.840	-1.63	1.69
